# Autoimmune neutropenia: a rare complication of allogeneic hematopoietic stem cell transplantation?

**DOI:** 10.3389/fimmu.2025.1640546

**Published:** 2025-07-24

**Authors:** María Isabel Benítez Carabante, Mariona Morell Daniel, María Luz Uría Oficialdegui, Enric Casanovas López, Melissa Panesso Romero, Laura Alonso García, Cristina Díaz-de-Heredia

**Affiliations:** ^1^ Division of Pediatric Hematology and Oncology, Hospital Universitari Vall d´Hebron, Barcelona, Spain; ^2^ Vall d´Hebron Institut de Recerca (VHIR), Barcelona, Spain; ^3^ Immunohaematology Laboratory, Banc de Sang i Teixits, Barcelona, Spain

**Keywords:** immune neutropenia, hematopoietic stem cell transplantation, children, graft failure, granulocyte antibodies

## Abstract

**Background:**

Autoimmune cytopenias (AIC) are rare complications of allogeneic hematopoietic stem cell transplantation (HSCT). Autoimmune neutropenia (AIN) is the least common type of AIC, and data on its incidence, risk factors and prognosis are scarce. This study aims to describe the incidence of AIN, its potential risk factors, and its clinical outcomes.

**Study design:**

This retrospective study included children who underwent a first allogeneic HSCT between 2015 and 2022. Patients with primary graft rejection were excluded. The primary endpoint was the incidence of AIN. Secondary endpoints included secondary graft failure (GF), overall survival (OS), and event-free survival (EFS).

**Results:**

A total of 208 patients were included, 30 of whom were diagnosed with AIN. The median time from HSCT to AIN diagnosis was 104 days, with a cumulative incidence of 8.73% (95%CI, 5.6-13.51) at 6 months and 14.74% (95%CI, 10.47-20.55) at 2 years after HSCT. No risk factors were found to be associated with AIN. The cumulative incidence of secondary GF at 2 years was 13.75% (95%CI, 5.38-32.68) in patients with AIN compared to 4.73% (95%CI, 2.39-9.25) in patients without AIN (p=0.06). There were no differences in terms of OS or EFS between patients with AIN and patients without AIN, with 3-year OS of 82.9% (95%CI, 63.6-92.5) vs 81.8% (95%CI, 75.26–86.77) (p=0.64) and 3-year EFS of 72.8% (95%CI, 52.8-85.4) vs 79% (95%CI, 72.2-84.31) (p=0.67). We identified two patients with specific human neutrophil alloantigen antibodies (anti-HNA), one of whom had a secondary graft failure.

**Conclusions:**

AIN may be a more frequent complication in post-HSCT pediatric patients than previously reported. Patients with AIN may be at a higher risk of secondary GF, but whether the risk of secondary GF is an important issue in patients with AIN needs to be explored in larger cohorts of patients. The study of specific anti-HNA in high-risk AIN patients should be considered.

## Introduction

Hematopoietic stem cell transplantation (HSCT) remains the curative treatment for both malignant and non-malignant diseases (1). Despite advances in prevention and supportive care, transplant-related complications continue to contribute to increased morbidity and mortality. In addition to the well-known complications of HSCT, the occurrence of autoimmune manifestations such as autoimmune cytopenias (AIC) has been described (2).

AIC is a rare and potentially life-threatening complication of allogeneic HSCT. However, early diagnosis and prompt treatment can improve patient outcomes (3). The reported incidence is variable, ranging from 1.5 to 6%, and has been reported as high as 22% in selected cohorts of pediatric patients with non-malignant diseases who have received HSCT from an alternative donor (4–8).

The pathophysiology of AIC is unclear but may be the result of impaired immune reconstitution and failure or loss of self-tolerance (9). Several mechanisms may contribute to the development of AIC: transfer of autoantibodies and reactive B and/or T cells from the donor to the recipient, impaired thymic function in the recipient, and reduced or dysfunctional regulatory T cells. AIC often develops during the tapering or withdrawal of immunosuppressive therapy but can occur from weeks to months after transplantation (10). The development of AIC is thought to involve multiple factors that contribute to immune dysregulation, including conditioning regimens, infections, graft-versus-host disease (GVHD), and the transfer of mismatched donor cells (7, 8). Reported associated risk factors include younger patient age, non-malignant diseases receiving an unrelated donor (UD) transplant, especially from cord blood (CB) (4, 5, 7, 8), and haploidentical donor. The use of lymphodepleting agents such as alemtuzumab, reduced intensity conditioning regimens, and viral infections such as cytomegalovirus (CMV), Epstein-Barr virus (EBV), and human herpesvirus-6 (HHV-6) (6) were also reported as risk factors (11).

AIC usually affects a single blood cell lineage but may also involve multiple cell lines. Autoimmune hemolytic anemia (AIHA) is the most common cytopenia observed, followed by immune thrombocytopenia (ITP) and autoimmune neutropenia (AIN) (3, 11, 12).

AIN after HSCT is very rare with only a few reported cases. In a study conducted by Kruizinga et al. AIN was observed in 9 (1.6%) of 531 transplants, alone or in combination with other AIC (6).

Clinical manifestations of AIN include fever and recurrent infections. It is characterized by a low absolute neutrophil count (usually <0.5×10^9^ cells per L) and the presence of anti-granulocyte antibodies. However, the sensitivity and specificity of these tests are limited (13, 14). In addition, other causes such as drug-induced neutropenia, disease relapse, graft failure (GF) and infection should be excluded.

To date, limited research has been published on AIN, and data on its incidence, risk factors, management and prognosis are scarce. The aim of this study is to describe the incidence of AIN after HSCT, to assess its clinical course and to evaluate current treatment strategies and treatment response. In addition, this study aims to explore potential risk factors associated with AIN, incidence of secondary GF and clinical outcomes.

## Materials and methods

### Study design

A single-center retrospective study conducted at the Pediatric Hematopoietic Stem Cell Transplant Unit of a tertiary care center. Children who underwent a first allogeneic HSCT and successfully engrafted between 2015 and 2022 were eligible for the study. Patients with primary GF were excluded. Data were collected from electronic medical records and patients were assigned a study number to ensure anonymity, with no identifiable information included in the database. Patients or their legal guardians signed an informed consent for data collection. The study was conducted in accordance with Good Clinical Practice guidelines, the Declaration of Helsinki, and the Spanish regulations on the protection of personal data. The research project was approved by the Ethics and Research Committee of the University Hospital of Vall d’Hebron (PR(AMI)472/2024). Clinical data collected included demographic information, patient and transplant characteristics, and post–transplant outcomes.

### Endpoints and definitions

The primary endpoint was the incidence of AIN. Secondary endpoints included secondary GF, overall survival (OS) and event-free survival (EFS), defined as the probability of being alive with sustained donor cell engraftment (death or secondary GF were considered events). Start time for all endpoints was the date of HSCT. Risk factors associated with AIN were analyzed.

AIN was defined as an absolute neutrophil count (ANC) of < 0.5 x10^9^/L in at least two determinations with a positive test for anti-granulocyte antibodies. The presence of anti-granulocyte antibodies was assessed by flow cytometry. A direct immunofluorescence assay was used to detect autoantibodies coating neutrophils, while the presence of anti-granulocyte antibodies in plasma was assessed by an indirect immunofluorescence test. Flow cytometry was performed using a CytoFLEX™ cytometer (Beckman Coulter), and data analysis was performed using CytExpert™ software (Beckman Coulter). A positive direct test was defined as a median fluorescence intensity (MFI) exceeding a previously validated laboratory cut-off. A positive indirect test was defined as an MFI at least twice that of a negative control (15–17).

Primary GF was defined as failure to achieve an ANC > 0.5 x10^9^/L (at day 30 for peripheral blood and bone marrow (BM) and at day 42 for (CB) with associated pancytopenia). Secondary GF was defined as a decline in hematopoietic function after having met the standard definition of hematopoietic recovery with secondary loss of donor cell engraftment (<10% of donor cell chimera) (18, 19).

Conditioning regimen intensity was classified according to the European Society for Blood and Marrow Transplantation (EBMT) definitions (20).

Acute GVHD was clinically graded according to the Mount Sinai Criteria (21) and chronic GVHD was diagnosed and graded according to the 2014 National Institute of Health consensus criteria (22).

### Statistical analysis

Quantitative variables are reported as median, range, and interquartile range (IQR). Categorial variables are reported as count and percentage. Patient age was considered a continuous variable. OS and EFS were calculated using the Kaplan-Meier estimator. Cumulative incidence was used to estimate the probabilities of AIN and secondary GF. Death was considered as a competing event. Probabilities are reported as percentage with 95% confidence interval (CI). Variables associated with AIN were evaluated in univariate analyses using a Cox proportional hazard model. Statistical analyses were performed with Stata.

## Results

### Patients and transplant characteristics

A total of 217 patients were transplanted during the study period, of whom 9 had a primary GF and were excluded from the study. Patient and transplant characteristics of the remaining 208 patients are summarized in **Table 1**. The median age at transplantation was 8 years. The distribution of underlying diseases was balanced between malignant and non-malignant diseases. UD and BM were the most common type of donor and graft source used. The most common conditioning regimen was myeloablative (MAC), with 27% of patients receiving total body irradiation (TBI). Most patients received anti-thymoglobulin (ATG) therapy. Nearly half of the patients developed acute GVHD.

**Table 1:** Patient and transplant characteristics of the 208 patients included.

Characteristic	Value
**Patients, n**	208
**Age at HSCT (years) median (range)**	8 (3.7-12.7)
Sex, n (%)	
Male	131 (63%)
Female	77 (37%)
Underlying disease, n (%)	
Malignant	98 (47%)
Non-malignant	110 (53%)
Donor type, n (%)	
Matched related donor	69 (32%)
Matched unrelated donor	117 (57%)
Haploidentical	22 (11%)
Graft source, n (%)	
Bone marrow	148 (71%)
Peripheral blood	36 (17%)
Umbilical cord blood	24 (12%)
Conditioning regimen, n (%)	
MAC / RTC	168 (80%)
RIC / nonmyeloablative	40 (20%)
Radiotherapy, n (%)	
Yes	57 (27%)
No	151 (73%)
Serotherapy, n (%)	
Yes	162 (78%)
• Alemtuzumab	15 (10%)
• Antithymoglobulin	147 (90%)
No	46 (22%)
Acute GVHD, n (%)	
Yes	91 (44%)
No	117 (56%)
Chronic GVHD, n (%)	
Yes	31 (14%)
No	177 (86%)
CMV infections, n (%)	
Yes	92 (44%)
No	116 (56%)

HSCT, hematopoietic stem cell transplant; MAC, myeloablative conditioning regimen; RTC, reduced toxicity conditioning regimen; RIC, reduced intensity conditioning regimen; GVHD, graft versus host disease; CMV, cytomegalovirus infection.

Thirty patients were diagnosed with AIN. Patient and transplant characteristics of those with AIN are shown in **Table 2**. The median age at transplantation was 8 years and 23 (77%) were male. The distribution of underlying diseases was balanced between malignant and non-malignant condition. Twenty-one patients (70%) received UD transplantation. MAC was the most common regimen (n=18; 60%), and serotherapy was used in 83% of patients. Grade II-IV acute GVHD was frequent (n=19; 63%). Most patients had a viral infection prior to the diagnosis of AIN, with CMV being the most common viral reactivation (n= 15; 50%).

**Table 2:** Patient and transplant characteristics of the 30 patients with autoimmune neutropenia.

Characteristic	Value
**Patients, n**	30
**Age at HSCT, (years) median (range)**	8 (0.3-18)
Sex, n (%)	
Male	23 (77%)
Female	7 (23%)
Underlying disease, n (%)	
Malignant	15 (50%)
• ALL	10 (33%)
• AML/MDS	5 (16%)
Non-malignant	15 (50%)
• Primary immunodeficiencies	9 (30%)
• Hemoglobinopathies	3 (10%)
• Bone marrow failures	2 (6%)
• Adrenoleukodystrophy	1 (3%)
Donor type, n (%)	
Matched donor	21 (70%)
• MUD	14 (46%)
• MSD	7 (23%)
Mismatched donor	9 (30%)
• MMUD	7 (23%)
• Haploidentical	2 (6%)
Graft source, n (%)	
Bone marrow	24 (80%)
Peripheral blood	5 (16%)
Umbilical cord blood	1 (3%)
ABO disparity, n (%)	
Yes	18(60%)
• Major	8 (26%)
• Minor	8 (26%)
• Bidirectional	2 (6%)
No	12 (40%)
Conditioning regimen, n (%)	
MAC	18 (60%)
RIC / non-myeloablative	12 (40%)
Radiotherapy, n (%)	
Yes	12 (41%)
• 12Gy	10 (33%)
• 2Gy	2 (6%)
No	18 (59%)
Serotherapy, n (%)	
Yes	25 (83%)
• Alemtuzumab	4 (13%)
• ATG	21 (70%)
No	5(17%)
GVHD prophylaxis	
CyA + MTX	19 (63%)
CyA + MMF	8 (27%)
CyA + MMF + PTCy	2 (7%)
CyA + Steroids	1 (3%)
Acute GVHD, n (%)	
Grade 0-I	11 (37%)
Grade II-IV	19 (63%)
Chronic GVHD, n (%)	
Yes	5 (17%)
No	25 (83%)
Viral infections, n (%)	
Yes	23 (77%)
CMV	15 (50%)
No	7 (23%)

HSCT, hematopoietic stem cell transplant; ALL, acute lymphoblastic leukemia; AML, acute myeloblastic leukemia; MDS, myelodysplastic syndrome; MUD, matched unrelated donor; MSD, matched sibling donor; MMUD, mismatched unrelated donor; MAC, myeloablative condition regimen; RIC, reduced intensity conditioning regimen; ATG, anti-thymoglobulin; CyA, cyclosporine A; MTX, methotrexate; MMF, mofetil mycophenolate; PTCy, post-transplant cyclophosphamide; GVHD, graft versus host disease; CMV, cytomegalovirus.

Of the 30 patients with AIN, one patient with X-linked hyper-IgM syndrome or CD40 ligand (CD40L) deficiency presented with pre-transplant AIN that persisted after transplantation. There have been 2 documented cases of AIN due to antibodies specifically directed against human neutrophil alloantigen (anti-HNA) 1a, previously reported (23), one in a 6-year-old boy with X-linked adrenoleukodystrophy (X-ALD) and the other in an 18-year-old girl with dyskeratosis congenita (DC). In both cases, the patients´ HNA-1a genotyping was negative, while the donor tested positive. The last three patients received a matched UD BM transplant.

### Cumulative incidence of AIN and risk factors

**Figure 1:**
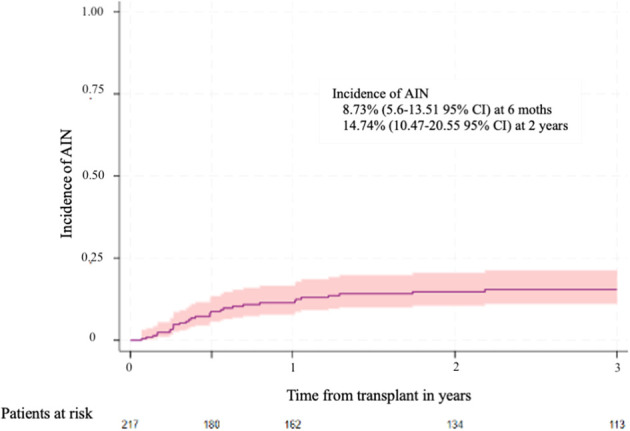
Cumulative incidence of autoimmune neutropenia. The 6 months and 2 years cumulative incidence of autoimmune neutropenia was 8.73% (5.6 – 13.51 95%CI) and 14.74% (10.47-20.55 95% CI), respectively.

The median time from HSCT AIN diagnosis was 104 days (IQR 22-170). The cumulative incidence of AIN at 6 months and 2 years after HSCT were 8.73% (5.6-13.51 95% CI) and 14.74% (10.47-20.55 95% CI), respectively (**Figure 1**).

The analysis of risk factors associated with AIN is shown in **Table 3**. No risk factors were found to be associated with AIN.

**Table 3:** Risk factors associated with autoimmune neutropenia.

Variable	Category	HR (95% CI)	p-value
Donor type	Unrelated donor	1	0.59
	Haploidentical donor	0.5 (0.12-2.17)	
	Related donor	0.79 (0.63-1.74)	
Graft source	PB	1.05 (0.43, 2.58)	0.36
	BM	1	
	CB	0.24 (0.03, 1.76)	
Conditioning regimen	MAC + RTC	1	0.7
	RIC	0.83 (0.32-2.17)	
Underlying disease	Malignant	1	0.87
	Non-malignant	0.94 (0.46-1.93)	
Serotherapy	No	1	0.83
	Yes	1.10 (0.45-2.7)	
*ex vivo* T cell depletion	No	1	0.74
	Yes	0.71 (0.1-5.24)	
Acute GVHD	No	1	0.74
	Yes	1.71 (0.83-3.52)	
Chronic GVHD	No	1	0.66
	Yes	0.79 (0.27-2.28)	
TBI	No	1	0.41
	Yes	1.38 (0.64-2.94)	
CMV infection	No	1	0.34
	Yes	1.41 (0.69-2.89)	
PID	No	1	0.54
	Yes	1.3 (0.56-3.03)	
SAA	No	1	0.12
	Yes	0.21 (0.03-1.55)	

HR, hazard ratio; CI, confidence interval; PB, peripheral blood; BM, bone marrow; CB, cord blood; MAC, myeloablative conditioning regimen; RTC, reduced toxicity condition; GVHD, graft versus host disease; TBI, total body irradiation; CMV, cytomegalovirus; PID, pediatric immunodeficiency; SAA, severe aplastic anemia.

### Infectious complications after AIN diagnosis

Thirteen patients (43.3%) had an infectious complication, of which 7 (54%) were considered severe and required admission to the intensive care unit. Four bacteremias were documented by Escherichia coli (n=2) and by Klebsiella pneumoniae (n=2). In addition, three invasive fungal infections (IFI) were reported: one hepatosplenic candidiasis, one probable pulmonary IFI, and one disseminated mucormycosis. The remaining 6 cases were classified as febrile neutropenia without microbiological documentation. These patients received antibiotics during the neutropenic and febrile periods, and all cases resolved without further complications.

### Other associated immune cytopenia

Twenty-three patients presented with AIN as isolated cytopenia (76.7%) and 7 patients in combination with other cytopenia (AIHA, n=4; ITP, n=2; AIHA and ITP, n=1).

### Treatment and response

All patients received granulocyte-colony stimulating factor (G-CSF) for a median time of 104 days (IQR 22-170). Patients with other cytopenias received other treatments including steroids, rituximab, and intravenous immunoglobulins (IVIG).

The CD40L-deficient patient had delayed neutrophil engraftment despite a MAC regimen and pretransplant rituximab. He received steroids, rituximab, and high-dose IVIG and was finally engrafted on day 40. He was later diagnosed with AIHA and ITP, from which he recovered after treatment, and at last follow-up was off immunosuppression with a stable mixed chimerism.

The 6-year-old boy with X-ALD failed to engraft neutrophils on day 30, with successful engraftment of the other cell lines. After antibodies against HNA-1a antigen were identified in the serum, the patient was treated with steroids, rituximab, and high-dose IVIG. Finally, neutrophil engraftment was achieved on day 54 after HSCT with complete donor chimerism. Unfortunately, the patient developed a severe pancytopenia and secondary GF 6 months after transplantation.

The patient with DC was diagnosed with ITP 2 years after transplantation and was successfully treated with a thrombopoietin receptor agonist. Three years after transplantation, persistent and severe neutropenia with recurrent infections was observed and she was diagnosed with AIN due to anti-HNA-1a. The patient was treated with rituximab and high-dose IVIG and is currently off immunosuppression with complete donor chimerism.

Overall, 24 patients (80%) had resolution of neutropenia and discontinued G-CSF, 1 patient responded but continued G-CSF at last follow-up, 1 patient died before response and 4 patients presented secondary GF.

### Secondary graft failure

**Figure 2:**
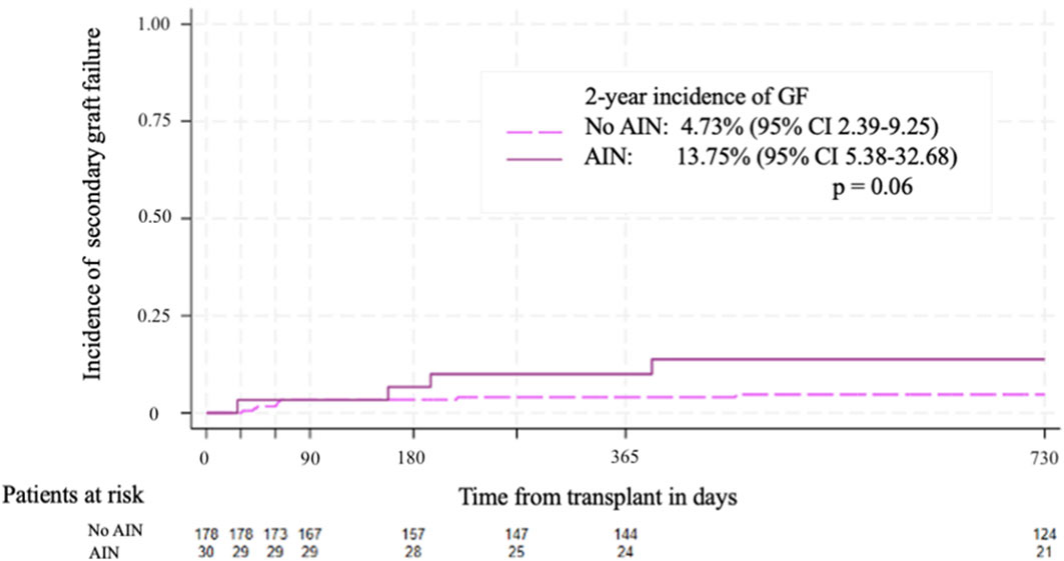
Cumulative incidence of secondary graft failure. The 2-year incidence of secondary graft failure was 4.73% (95%CI 2.39 – 9.25) for patients without AIN and 13.75% (95%CI 5.38 – 32.68) for patients with AIN, p=0.06.

A total of 4 patients in the AIN cohort presented with secondary GF after the diagnosis of AIN, including the 6-year-old boy with X-ALD and anti-HNA-1a. The cumulative incidence of secondary GF at 2 years in patients with AIN was 13.75% (95% CI 5.38-32.68) compared to 4.73% (95% CI 2.39-9.25) in patients without AIN (p=0.06) (**Figure 2** **).**

The characteristics of the 4 patients with secondary GF are detailed in **Table 4**. Most patients had a non-malignant disease and had received an unrelated donor transplant. All patients had received bone marrow as the graft source and serotherapy. Patients did not have acute or chronic GVHD. All four patients who experienced GF received a second transplant and engrafted successfully, and three were alive at last follow-up. The X-ALD patient died due to progressive cerebral leukodystrophy.

**Table 4:** Characteristics of the 4 patients with autoimmune neutropenia and secondary graft failure.

Patient	Age	Underlying disease	Year of HSCT	Graft source/ donor	Conditioning regimen and serotherapy	GVHD	Viral infections prior to AIN diagnosis	Time from HSCT to AIN diagnosis (days)	Time from AIN diagnosis to GF (days)	Donor and graft source of 2^nd^ HSCT	Status
1	6y	X-linked adrenoleukodystrophy	2018	BM MUD	Bu*Flu ATG	No	EBV	25	156	BM MUD	Died due to disease progression.
2	15y	B-acute lymphoblastic leukemia	2019	BM MMUD	VP16,TBI 12Gy ATG	No	CMV	88	69	BM Haploidentical	Alive AIN free
3	9a	Chronic granulomatous disease	2019	BM MUD	Bu^+^ Flu ATG	No	No	19	30	BM Haploidentical	Alive AIN free
4	3m	PID (Leukocyte adhesion deficiency)	2019	BM MRD	FluMel Alemtuzumab	No	No	70	60	BM MRD^#^	Alive AIN free

HSCT, hematopoietic stem cell transplantation; GVHD, graft versus host disease; AIN, autoimmune neutropenia; GF, graft failure; BM, bone marrow; MUD, matched unrelated donor; BuFlu, busulfan and fludarabine; ATG, anti-thymoglobulin; EBV, Epstein Barr virus; MMUD, mismatched unrelated donor; VP16, etoposide; TBI total body irradiation; CMV, cytomegalovirus; PID, pediatric immunodeficiency; MRD, matched related donor; FluMel, fludarabine and melphalan.

*AUC 85–95 mg*h/L.

+AUC 65–75 mg*h/L.

# Same donor and graft source as in the first transplantation.

### Follow up, survival and causes of death

**Figure 3:**
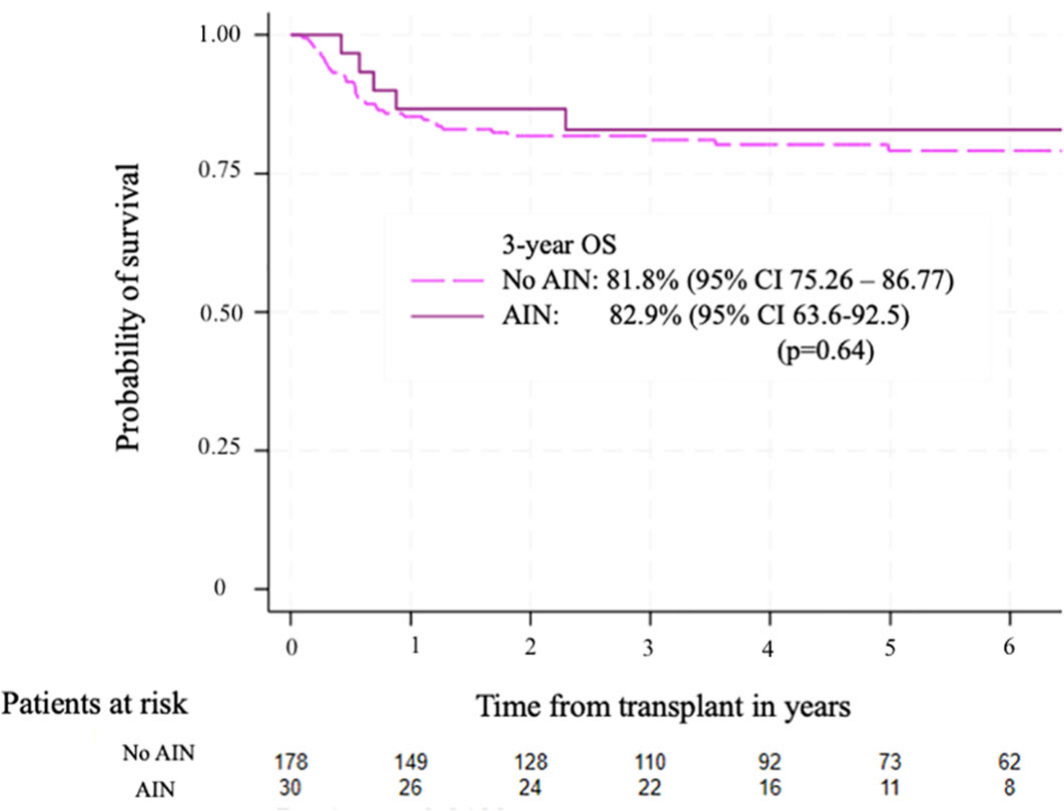
Overall survival according to the presence of autoimmune neutropenia. The 3-year OS was 81.8% (95%CI 75.26-86.77) for patients without AIN and 82.9% (95% CI 63.6 – 92.5) for patients with AIN, p=0.64.

**Figure 4:**
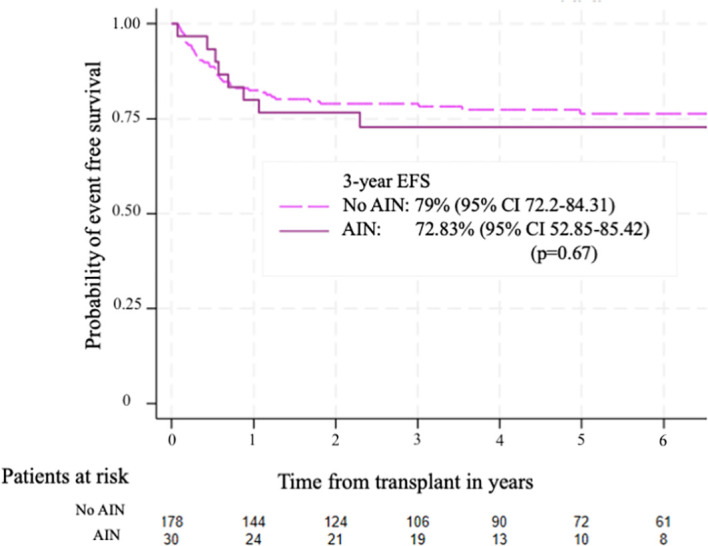
Event free survival according to the presence of autoimmune neutropenia. The 3-year EFS was 79% (95%CI 72.2-84.31) for patients without AIN and 72.83% (95%CI 52.85 – 85.42) for patients with AIN, p=0.67.

The median follow-up after transplantation was 29 months (IQR 18-48). The 3-year OS for patients with AIN was 82.9% (95% CI 63.6-92.5), while the 3-year OS for patients without AIN was 81.8% (95% CI 75.26 – 86.77) (p=0.64) **Figure 3**. The 3-year EFS was 72.8% (95% CI 52.8-85.4) for patients with AIN and 79% (95% CI 72.2-84.31) for patients without AIN (p=0.67) **Figure 4**.

There were 5 deaths in the AIN group (17%), 1 due to a serious infection (mucormycosis) associated with severe neutropenia, and the remaining deaths were not related to AIN (3 patients died of underlying disease progression and 1 of diffuse alveolar hemorrhage).

## Discussion

This single-center retrospective study presents the outcomes of AIN in 30 pediatric patients following HSCT and provides real-world clinical data, focusing on the characterization of these patients, AIN associated complications, treatment response, and survival.

AIN has been described as a rare immunologic complication after HSCT with a low incidence reported. However, the 3-year cumulative incidence of AIN in our cohort was significantly higher, at 15.4%. This may be due to the characteristics of the patients included in the study, a pediatric population in which half of the patients had a non-malignant disease, 60% receiving an UD transplant, in addition to the 80% of the patients receiving serotherapy and a high incidence of viral infections. All these factors have been previously associated with the development of other AIC (11, 24). Furthermore, in recent years, we have included anti-granulocyte antibody screening in post-transplant patients who presented with persistent or severe neutropenia (ANC < 0.5 x10^9^/L). Increased awareness of AIN may have contributed to the higher number of patients diagnosed. However, it is important to rule out other potential causes of post-transplant neutropenia in this context, such as a relapse of the underlying disease, infections causing transient leukopenia, or the use of myelotoxic drugs.

Diagnosis of AIN can be challenging. Anti-granulocyte antibodies may be present on testing, but their presence does not exclude other causes of these neutropenia. On the other hand, their absence does not exclude a diagnosis of AIN (24), as anti-granulocytes antibodies may be transiently absent. Conversely, multiple blood transfusions can result in the development of anti-granulocyte antibodies but the incidence of alloimmunization has been significantly reduced by current transfusion protocols. These protocols include the universal leukoreduction of blood products and reducing the amount of donor plasma in the products by replacing it with synthetic solutions (25).

Flow cytometry immunofluorescence analysis remains the gold-standard technique for detecting anti-granulocyte antibodies (16). However, a key limitation of this analysis is the lack of harmonization in defining a positive result. For the direct test, positive results are interpreted based on an MFI value threshold validated according to the direct fluorescence test technique, which is read by optical microscopy - an observer-dependent method. Similarly, there is no established cut-off point for the indirect test. The assay requires granulocytes obtained from blood donors as test reagents, and there is no reference standard to normalize the results. Without a harmonized definition of a positive result, comparability and reproducibility across centers may be compromised. In addition, there is no lineal correlation between fluorescence intensity and antibody title, and the degree of neutropenia reflects a balance between antibody consumption and bone marrow production.

Another limitation from flow cytometry immunofluorescence analysis is its inability to determine their specificities. Antibody characterization in AIN is usually reserved to selected cases, such as suspected neutrophil GF or a sustained neutropenia caused by donor-specific granulocyte antibodies (26), given the high cost and/or time-consuming nature of additional complementary techniques. One way to determine this would be to use Luminex techniques with the LABSCreen™ kit, but this would significantly increase the economic and personnel costs of these studies (27).

The time from transplantation to the AIN onset may be variable, and although in our cohort most patients were diagnosed in the first year after transplantation, AIN can occur at any time during the follow-up. In other retrospective reports, AIN was often associated to other cytopenia (6, 10, 28, 29), however, in our retrospective study, 77% of patients were diagnosed with isolated AIN. On the other hand, some patients may present with pre-transplant AIN that may recur or persist after HSCT, such as in the case of patients with inborn errors of immunity (30). In these patients, screening for the presence of AICs and actively treating them prior to initiating the conditioning regimen may improve transplant outcomes.

One of the aims of our study was to identify risk factors that might be associated with AIN in the post-transplant period. However, we were not able to identify any statistically significant risk factors. A possible explanation was the small number of patients included in the analysis and the limited number of events. Continued patient recruitment and an increase in sample size may allow the identification of risk factors associated with the occurrence of AIN. Twenty percent of patients with AIN presented with ABO incompatibility but we were not able to evaluate it as a risk factor due to missing data in the cohort of patients who did not developed AIN. There is little evidence to support a direct role regarding its involvement in the development of neutropenia. Unlike platelets, neutrophils do not express ABO antigens (31). However, shared antigens have recently been identified between erythrocytes and neutrophils, specifically within the granulocyte HNA-3 system and the Cost antigen of erythrocytes (32). This could supposedly produce neutropenia due to increased immunization in sensitized individuals.

In our cohort, most patients had received serotherapy with ATG. Although ATG is an immunosuppressive agent derived from animals, its role in inducing anti-granulocyte antibodies is controversial. It is mainly composed of polyclonal antigens that act against T-cell surface antigens (such as CD2, CD3, CD4 and CD8), which are absent from the surface of granulocytes. Transient cytopenias may occur during ATG therapy, but this is more likely due to the immunosuppressive effects of ATG than to a sustained humoral response against granulocytes. Conversely, although some patients, particularly those who have received repeated administrations, may develop xeno-antibodies, this immune response is neither consistent nor necessarily clinically significant (33).

**Figure 5:**
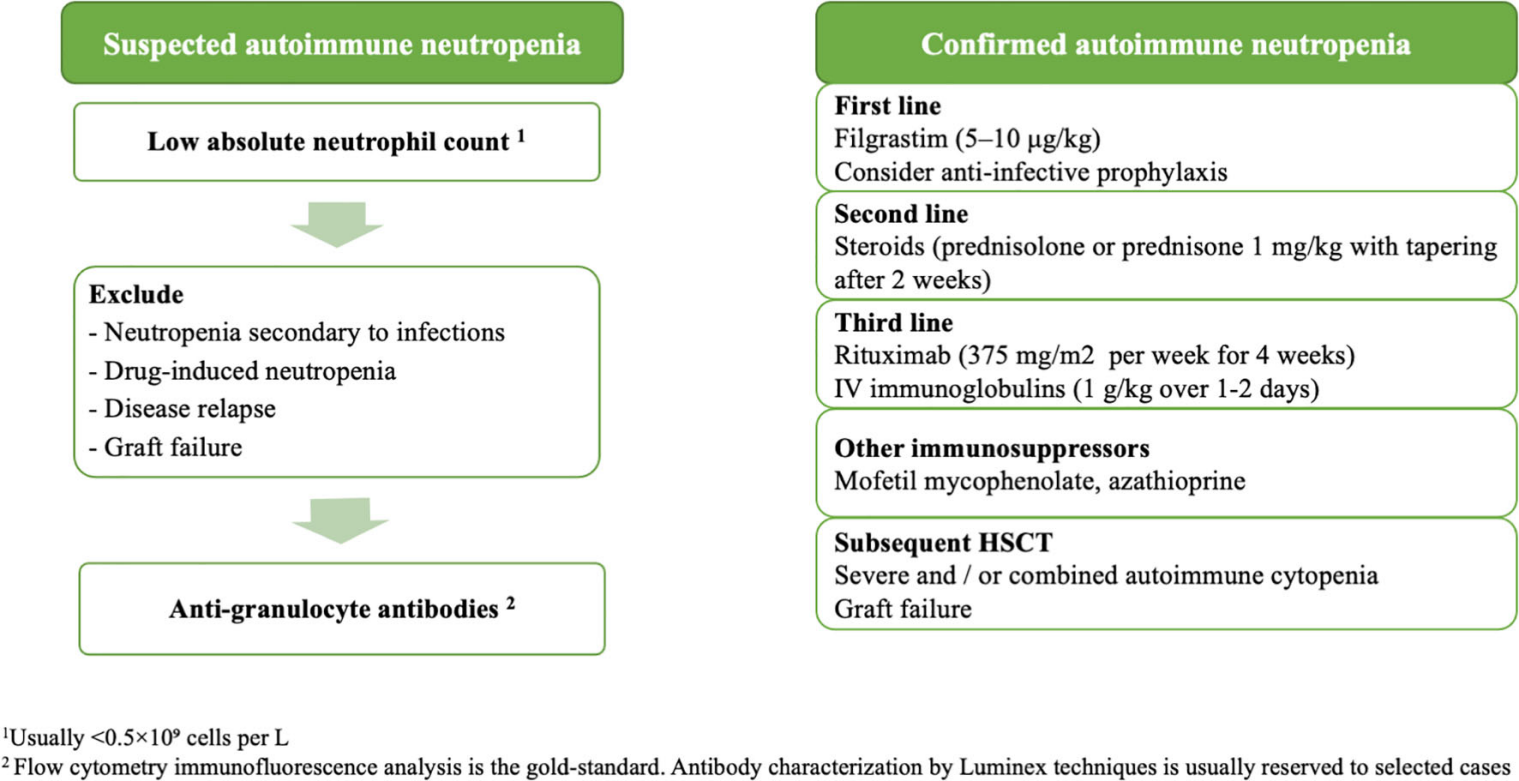
Suggested algorithm for the diagnosis and management of immune-mediated neutropenia in patients who have undergone allogeneic hematopoietic stem cell transplantation. Diagnosis of AIN: AIN should be suspected in patients who have undergone HSCT and present with a low ANC. In addition to the study of anti-granulocyte antibodies, other causes of neutropenia should be investigated. Flow cytometry immunofluorescence analysis remains the gold standard technique for detecting anti-granulocyte antibodies. Antibody characterization by Luminex techniques is usually reserved to selected cases, such as suspected neutrophil graft failure or a sustained neutropenia caused by donor-specific granulocyte antibodies. Treatment of AIN: Treatment options include filgrastim, corticosteroids, intravenous immunoglobulins, rituximab and other immunosuppressor agents. Furthermore, patients with severe AIN should receive antifungal prophylaxis.

There is no clear consensus on how to manage post-transplant AIN. In our cohort, all patients received G-CSF. Other treatment options were reserved for those patients with additional cytopenias, or with persistent or refractory neutropenia. This was the case for the two patients with specific anti-HNA antibodies and the patient with CD40L deficiency. The treatment used in these cases was similar to that reported by other groups, involving the use of steroids and high-dose IVIG or rituximab in cases of severe and refractory neutropenia (6, 10, 11, 24, 34, 35). Other immunosuppressors used to treat other AIC, such as mycophenolate mofetil and azathioprine, may be considered. Based on current practices summarized by experts in the field (10, 11), as well as our own clinical experience, we have proposed an algorithm for the treatment and management of AIN after HSCT (**Figure 5**).

In our cohort, two patients had delayed neutrophil engraftment and four patients had secondary GF. Although the difference was not statistically significant, a higher incidence of secondary GF was observed in patients with AIN, with a 13.75% (95% CI 5.38-32.68) compared to 4.73% (95% CI 2.39-9.25) in patients without AIN (p=0.06). One of the patients with anti-HNA-1a had a secondary GF. While research on the role of anti-HNA is limited, recent publications highlight the potential risk between HNA antibodies and transplant outcomes, showing that anti-HNA may affect the time to neutrophil engraftment and increase the risk of secondary GF (26).

Although there were no differences in terms of OS and DFS in patients with AIN and patients without AIN, 23% of patients presented with severe infection and required intensive care support and 1 patient died due to an IFI. This fact underlines the need for antifungal prophylaxis in all transplanted patients with severe neutropenia. Although the use of antibacterial prophylaxis, except for anti-pneumococcal prophylaxis, is controversial, an early admission and initiation of broad-spectrum antibiotics in patients with fever and severe neutropenia should be guaranteed.

The limitations of our study include its retrospective design, single-center nature, and small sample size. In fact, only 30 cases of AIN were identified in a cohort of 208 transplants, which reduces the statistical power of the study.

In conclusion, AIN may be a more common complication in pediatric patients undergoing HSCT than previously reported, and it should be suspected in cases of persistent or severe neutropenia. While AIN does not appear to have an impact on survival, some patients may present with serious, life-threatening infections. Patients with AIN may be at an increased risk of delayed engraftment and secondary GF, but whether the risk of secondary GF is an important issue needs to be evaluated in larger cohorts of patients. Finally, the characterization of specific anti-HNA antibodies in high-risk AIN patients should be considered.

## Data Availability

The original contributions presented in the study are included in the article/supplementary material. Further inquiries can be directed to the corresponding author.
